# Contrasting effects of male immigration and rainfall on rank-related patterns of miscarriage in female olive baboons

**DOI:** 10.1038/s41598-021-83175-3

**Published:** 2021-02-17

**Authors:** Andrea Bailey, Lynn E. Eberly, Craig Packer

**Affiliations:** 1 Ecology, Evolution, and Behavior, University of Minnesota, St. Paul, MN 55108 USA; 2 Biostatistics, University of Minnesota, Minneapolis, MN 55455 USA; 3 Biology, Phillips Academy, Andover, MA 01810 USA

**Keywords:** Behavioural ecology, Sexual selection, Animal behaviour

## Abstract

In mammalian species with prolonged maternal investment in which high-ranking males gain disproportionate numbers of mating opportunities, males that quickly ascend the hierarchy may benefit from eliminating the dependent offspring of their competitors. In savanna baboons, high-ranking females are the most profitable targets of infanticide or feticide, because their offspring have higher survival rates and their daughters reach sexual maturity at a younger age. However, such patterns may be obscured by environmental stressors that are known to exacerbate fetal losses, especially in lower-ranking females. Using 30 years of data on wild olive baboons (*Papio anubis*) in Gombe National Park, Tanzania, we found evidence that rapidly-rising immigrant males induced miscarriages in high-ranking females outside of drought conditions. However, miscarriage rates were largely reversed during prolonged periods of low rainfall, suggesting that low-ranking females are particularly vulnerable to low food availability and social instability. Infanticide did not emerge as a recurrent male strategy in this population, likely because of the protective behavior of resident males towards vulnerable juveniles.

## Introduction

Male mammals can accelerate the return of lactating females to a receptive state by killing their unweaned offspring ^1,2^. Pregnant females carrying the offspring of rival males may also be targeted for aggression, as induced abortions can confer similar benefits to incoming males ^3–6^. However, in social groups where multiple males compete for mating access and top-ranking individuals gain priority of access to estrous females, only a subset of males might benefit from such strategies.

Spontaneous pregnancy loss, often referred to as the “Bruce effect”, can be adaptive where females would otherwise lose their forthcoming offspring to infanticide by immigrating males ^6–10^. But in many primate species, resident males often remain after the arrival of new males and can protect their offspring from infanticide ^11,12^. However, incoming males would still benefit by deliberately inducing miscarriage during vulnerable stages of pregnancy ^5^. In savanna baboons, the incidence of infanticide varies widely, occurring frequently in chacma baboons but rarely in olive baboons ^12,13^. Since male chacma baboons ascend rank quickly, incoming males are likely to father the offspring of any females that resume estrous during their first months in the troop ^12–14^. Olive baboons typically attain rank more slowly ^13^, and reproductive skew is less pronounced, with lower-ranking males utilizing alternative mating strategies ^15–17^. Thus, the benefits of infanticide/feticide would largely be restricted to individual olive males that follow a rapid-rising trajectory similar to a chacma male, and feticide would circumvent the protective behavior of care-taking resident males that frequently handle or carry small infants ^11,18^. Alberts et al*.* ^19^ documented a particularly aggressive male immigrant that attained top rank within a week of his arrival as well as three associated fetal losses accompanied by high cortisol levels in females that suggests such a strategy is feasible and likely present in savannah baboons.

In multi-female primate groups, the offspring of dominant females enjoy higher survival and earlier maturation of daughters ^20,21^, thereby providing fitness advantages for fathers. Thus, males that are able to rapidly attain high rank should preferentially target high-ranking pregnant females. Fetal loss in response to male take-overs has been documented in several mammalian taxa ^3,5,7,10,22^, and recent work in yellow baboons highlights the importance of male rank ^5^, though there remains little evidence that males preferentially direct their efforts toward specific females. Here, we utilize long-term data from Gombe National Park in Tanzania, to test whether previously reported rank-related differences in miscarriage rates in this population ^20^ are driven by social factors relating to the arrival of new rapidly-rising males, taking into account year-to-year variations in environmental stress. More specifically, we test whether, first, high-ranking pregnant females exposed to rapidly-rising immigrant males are more likely to miscarry relative to their unexposed counterparts, and, second, if their offspring are similarly vulnerable to infanticide. As females may be wounded during the males’ efforts to induce miscarriage, we assess whether pregnant females are wounded at higher rates following the entrance of rapidly-rising males. Finally, because environmental stress can also lead to miscarriage ^23^, we evaluate the role of rainfall as a proxy for food abundance and its impact on pregnancy.

## Results

**Figure 1:**
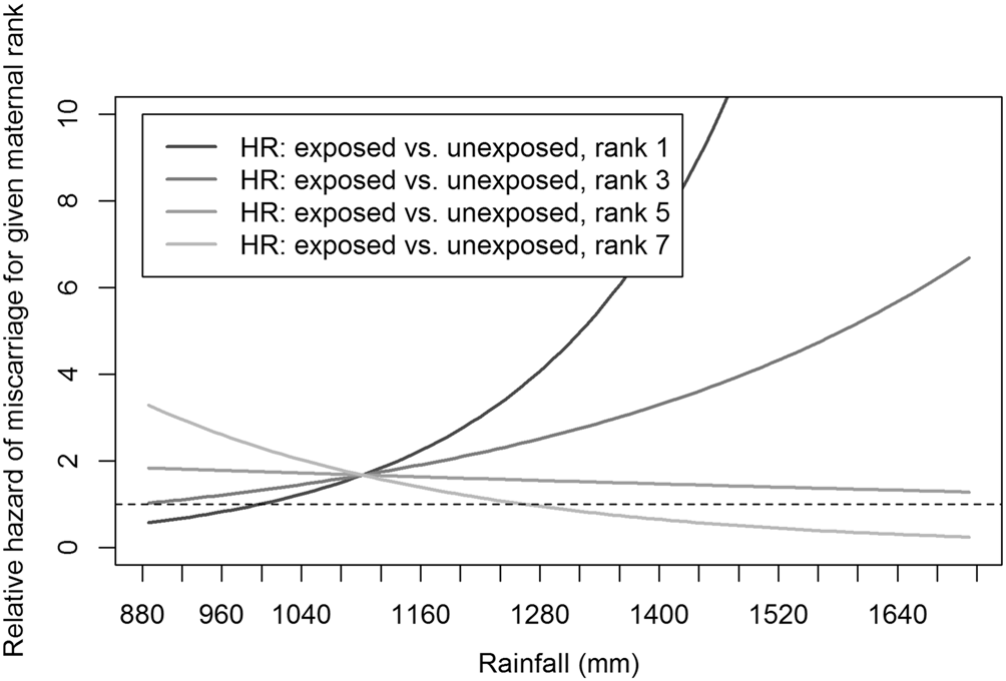
The hazard of miscarriage of pregnant females exposed to immigrant males that ascended to top rank within 1 year relative to unexposed pregnant females (n = 732 pregnancies; Cox regression with time-dependent covariate: 3-way interaction, p = 0.008, two-tailed). The plot includes hazards for females of rank 1, 3, 5, and 7 relative to unexposed like-ranked counterparts. All values are calculated across the known range of 2-year mean rainfall at Gombe National Park.

We found a strong interaction between exposure to males that rapidly attained top rank, female rank and average 2-year rainfall on miscarriage rates (3-way interaction, p = 0.008; Table 1, Fig. 1). Specifically, outside drought conditions, higher-ranking pregnant females exposed to soon-to-be alpha males had a higher hazard of miscarriage than both their non-exposed counterparts and lower-ranking females, and this effect was exacerbated by increasing rainfall. However, the model shows that the effect of maternal rank essentially disappeared when the average amount of rain fell below ~ 1100 mm with all females suffering similar levels of fetal loss in response to incoming males. Note that the 3-way interaction remains highly significant (p = 0.003) when controlling for female age (which has a strong effect on miscarriage rates in this population)^20^. Also note that these patterns only held for pregnant females exposed to rapidly-rising males; females exposed to new immigrants as a whole had a similar hazard of miscarriage as unexposed individuals, regardless of rank or rainfall (HR 1.30, 95% CI (0.771, 2.178), p = 0.33; Table 1). Interestingly, we found little evidence for infanticide perpetrated by these top males (effects on infant survival: HR 0.864, 95% CI (0.651, 1.146), p = 0.31; Table 1).

**Table 1:** Cox regression results for time-dependent covariate analyses of the hazard of: (1) miscarriage for pregnant females (Fetus) and (2) death prior to 1 year of age for infants (Infant) when exposed to any new immigrant male (All new immigrants) or only new immigrant males that achieved the top rank in the group within 1 year of their immigration (Rapid-rising new immigrants).

Covariates	HR	All new immigrants		Rapid-rising new immigrants		
		95% CI	*P*	HR	95% CI	*P*
**Fetus (n = 732)**						
Exposure	1.298	0.761–2.216	0.338	1.457	0.572–3.712	0.430
Exposure × maternal rank	0.988	0.850–1.148	0.875	0.936	0.814–1.077	0.360
Exposure × 2-year avg. rainfall	1.000	0.997–1.003	0.967	0.997	0.993–1.001	0.150
Exposure × maternal rank × 2-year avg. rainfall	1.000	0.999–1.001	0.784	0.999	0.998–0.999	**0.008**
**Infant (n = 681)**						
Exposure	1.216	0.839–1.762	0.302	0.888	0.664–1.188	0.423
Exposure × maternal rank	1.030	0.947–1.12	0.488	0.980	0.922–1.041	0.508
Exposure × 2-year avg. rainfall	0.999	0.998–1.001	0.412	1.000	0.999–1.002	0.728
Exposure × maternal rank × 2-year avg. rainfall	1.000	0.999–1.000	0.160	1.000	1.000–1.001	0.822

*HR* hazard ratio, *CI* confidence interval.

All significant results are bolded.

**Figure 2:**
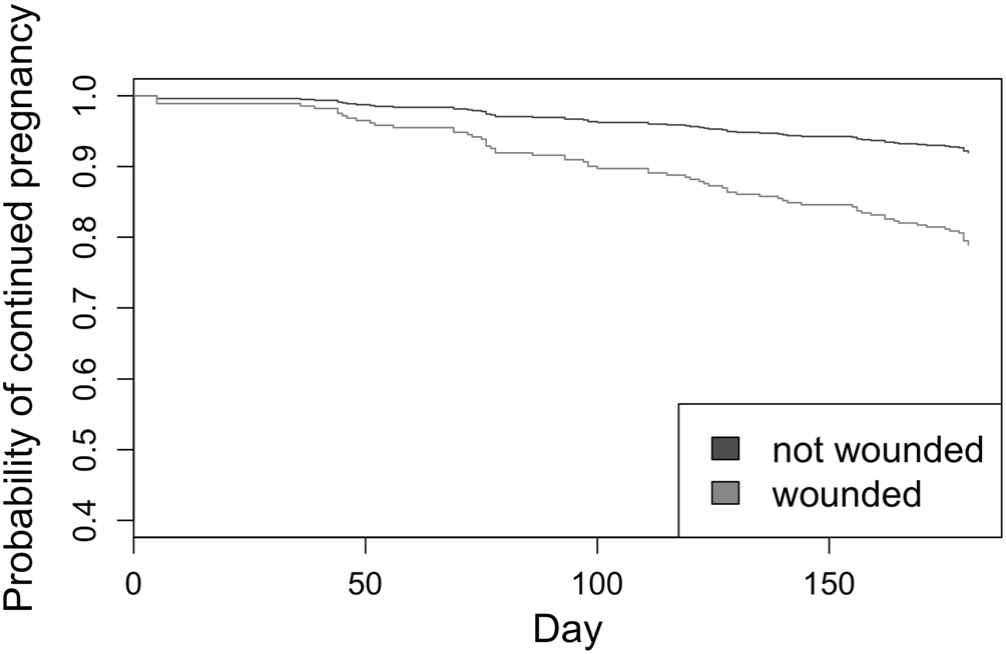
The probability of continued pregnancy for females wounded during pregnancy vs. those not wounded (n = 768 pregnancies with 71 involving at least one wound; Cox regression with time-dependent covariate: hazard ratio (HR) 2.83, 95% CI (1.40, 5.72), p = 0.004, two-tailed).

Adult baboons at Gombe have virtually no predators, thus any injuries result from within-species aggression ^24^. Females wounded during pregnancy suffered higher hazards of miscarriage (HR 2.83, 95% CI (1.40, 5.72), p = 0.004; Fig. 2), and pregnant females suffered a disproportionate number of wounds after the arrival of a male that quickly ascended to top rank as compared to unexposed pregnant females (proportion expected: 8.6%, proportion observed: 24.7%, n = 768; x^2^ = 24.29, p < 0.00001). Note, however, that there were too few wounds to test for effects of female rank.

## Discussion

Our results corroborate evidence from other baboon species that rapidly-rising males utilize feticide as a reproductive strategy. However, the males at Gombe largely do not regularly engage in infanticide and largely restrict their feticidal behavior to high-ranking females during years with favorable rainfall. Given that rank plays such an important role in female reproductive performance, males that mate with high-ranking females accrue increased fitness. Previous work on the Gombe baboons showed that top-ranked females suffered a cost of rank due to a disproportionately high miscarriage rate ^20^. Our results suggest that high-ranking females suffer reduced fitness exactly because their offspring enjoy higher survival and earlier maturation, making them ideal targets for competitively superior males.

The lack of evidence for infanticide, even in apparently feticidal males, suggests that the benefits of feticide are not only higher than infanticide but that olive baboons have developed more effective counterstrategies to the latter. Zipple et al.^6^ highlight that pregnant females are further from their next conception than lactating females, and thus males that do not commit feticide suffer higher reproductive losses than males that do not commit infanticide. Feticide may then be the more strongly selected behavior as well as easier to execute. Infanticide necessarily requires the wresting of an infant from its mother, likely in the proximity of its father, which can be both difficult and dangerous. Inducing miscarriage may only require a few stressful chases or one successful bite. Though fathers may still be protective in this context ^25^, the interaction may be more easily executed at any given moment, whereas infanticide may involve brief windows of opportunity when the infant is not on its mother or being carried by a resident male ^11,12,14,18^. Also, a fetus may simply be less valuable to potential fathers than a 1–12 month old infant.

The disproportionate number of injuries among pregnant females after the arrival of rapidly-rising males imply that these males employ specific aggressive behaviors to induce miscarriage, though further observational evidence are needed to confirm whether they selectively target high-ranking females. Whereas conspecific-inflicted injuries provide a clear mechanism for inducing miscarriage via the release of acute stress hormones and bacterial infection, chronic low-grade stress can also lead to fetal loss in mammals ^26–29^. During times of food scarcity, low-ranking females exposed to rapidly rising males suffered similar rates of miscarriage relative as high-ranking females. New immigrant males often destabilize troops upon their arrival ^30^, and males that rapidly rise to top rank may disrupt group dynamics most of all. Thus, low-ranking females may miscarry in response to generalized social instability when coupled with intense feeding competition, thereby masking any effects from female rank. The arrival of fast-rising males at Gombe has a striking impact on the pregnancies of high-ranking females during periods of plenty, suggesting a new twist to the Battle of the Sexes, where the “best” males (those capable of a rapid initial rise in dominance rank) may focus their most destructive behavior on the “best” females (those with the fastest reproductive rates). The intersection of rank, environmental stress, and exposure to new males emphasizes the value of adopting a more nuanced evaluation of the reproductive conflicts between males and females.

## Methods

### Study area and population

Located on the eastern shore of Lake Tanganyika in Tanzania, Gombe National Park is approximately 52 km^2^ and is characterized by small streams and steep valleys that descend from a rift escarpment. Gombe lacks large wild predators ^31^ and is home to several primate species, including olive baboons, chimpanzees, and red colobus monkeys. Baboons at Gombe have been observed since 1967, with continuous demographic data collected since 1972. Data were collected on two distinct study groups, which subsequently split several times throughout the years, yielding a total of 9 troops with a mean size of 22.1 (± 0.3 SE) adults, including 7.0 (± 0.08 SE) adult males and 14.2 (± 0.2 SE) adult females. These data include daily reproductive status of females, immigration and emigration events, births, and deaths. Male and female ranks were determined using the outcomes of pairwise interactions between same-sex individuals, with those exhibiting more submissive behaviors in repeated encounters ranked below the individual to which they were submissive.

### Miscarriage data

Birth and miscarriage data are available from 1972 to 2002. Pregnancy in females is identified by the appearance of bright red coloration on the paracollosal skin of the rump ^32^. This color change is overt, appearing approximately 3 weeks after the sexual swelling begins to detumesce ^25^, and persists until several days after parturition, providing a reliable indicator of reproductive status ^33^. Gestation typically lasts 180 days ^34^. Vaginal bleeding by pregnant females and the complete loss of pregnancy coloration were used to estimate fetal age at miscarriage. The underlying miscarriage rate for Gombe baboons is 10.7%, though this is likely an underestimate because miscarriages during the first three weeks of pregnancy cannot be detected from visual cues. Full-term stillborn’s were not considered to have been miscarried.

### Miscarriage and infanticide analyses

#### Fetal exposure to immigrant males

Fetal age at first exposure to a new immigrant male with known rank 1 year from immigration was determined for all pregnancies of females with known rank (n = 732; 175 total females; 65 pregnancies resulted in miscarriage). A Cox regression with a time-dependent covariate was used to calculate hazard of miscarriage to fetal age of 180 days, with exposure to immigrant male updated to “yes” at the age of a fetus’s first exposure to a new immigrant; a cluster effect was used to account for multiple pregnancies for the same female. A second analysis classified pregnant females by exposure to a male that ascended to top rank within 1 year of immigration, with only those pregnancies classified as “exposed” at the fetal age of first exposure. A significant decrease in rainfall at Gombe over several decades introduced an increasing environmental stressor to females—drought has been shown to increase rates of miscarriage in yellow baboons ^23^ and rain-mediated food abundance also impacts seasonal reproduction in geladas ^35^—and so both 2-year rainfall means and maternal rank were included as predictors in these regressions. Other predictors, such as group size and maternal age were evaluated in a previous analysis ^20^. Though age remained the only other significant predictor of miscarriage, its inclusion did not change the 3-way interaction result and thus was not included in the final Cox regression analysis.

#### Infant exposure to immigrant males

Infant age at first exposure to a new immigrant male with known rank 1 year from immigration was determined for all infants from birth to 1 year of age (n = 681). A Cox regression with a time-dependent covariate was used to calculate hazard of death to infant age of 1 year, with exposure to immigrant male updated to “yes” at the age of an infant’s first exposure to a new immigrant. No distinction was made between infants exposed to one male and those exposed to multiple males; the earliest exposure age was used. A second analysis classified infants by exposure to a male that ascended to top rank within one year of immigration, with only those infants classified as “exposed” at the age of first of exposure. Maternal rank and 2-year rainfall means were also used as a predictor in these regressions.

A total of 233 male immigrants with known rank were used in these analyses, with 30 males ascending to top rank within a year of immigration.

### Wounding analysis

Wounds are defined as a visible injury anywhere on the animal’s body. Though varying in severity, we treated all wounds identically. Fetal age at each wounding event was determined for all pregnancies; unwounded females were classified as having 0 wounds during pregnancy and this score was updated to 1 at the fetal age of the wounding event. A Cox regression with a time-dependent covariate was then used to calculate the hazard of miscarriage before day 180 for wounded vs. non-wounded pregnant females (n = 768 pregnancies; 71 pregnancies involved at least one wound), controlling across maternal rank; a cluster effect was used to account for multiple pregnancies for the same female. A chi-square analysis was also used to determine whether a disproportionate number of pregnant females were wounded immediately after the arrival of a rapid-rising male as compared to expected. Expected values were calculated using the total number of pregnant females wounded (n = 71) out of the total number of females wounded (n = 828). Out of 768 pregnancies, 77 were exposed to males that attained top rank within a year.

All analyses were carried out in R version 3.1.1. All reported p-values are two-tailed, with p < 0.05 considered statistically significant.

### Statement of ethics

As a naturalistic observational study, no IACUC permits were required.

## Data Availability

The data that support the findings of this study are available from the corresponding author upon reasonable request.
